# Clinical outcomes and determinants of neonatal hyperbilirubinemia among NICU admissions: a retrospective study in Ethiopia

**DOI:** 10.1038/s41598-026-55805-1

**Published:** 2026-06-12

**Authors:** Bacha Gishu, Tesfa Gebremeskel, Ayalneh Demissie, Girma Worku, Tahir Aman, Addis Wordofa

**Affiliations:** 1 Department of Pediatrics, College of Health Sciences, Arsi University, Asella, Ethiopia; 2 Department of Public Health, College of Health Sciences, Arsi University, Asella, Ethiopia; 3 Department of Anaesthesia, College of Health Sciences, Arsi University, Asella, Ethiopia

**Keywords:** Neonatal jaundice, Ethiopia, Neonatal sepsis, Inadequate breastfeeding, Neonatal hyperbilirubinemia, Clinical outcomes, NICU, ARTH, Diseases, Health care, Medical research, Risk factors

## Abstract

Neonatal hyperbilirubinemia is a significant public health concern in Ethiopia, contributing to approximately 10% of neonatal mortality. This study aimed to identify the clinical outcomes and determinants of jaundice among neonates admitted to the Neonatal Intensive Care Unit (NICU) at Asella Referral and Teaching Hospital (ARTH) in South East Ethiopia. The objective of this study was to assess the magnitude of neonatal hyperbilirubinemia, its clinical outcomes, and the maternal and neonatal determinants among neonates admitted to the ARTH NICU. An institution-based retrospective cross-sectional study was conducted using systematic random sampling (K = 7), 183 neonates were selected randomly from a source population of 1,350 admissions. Data were analyzed using multivariate logistic regression to identify independent predictors of hyperbilirubinemia, with statistical significance set at *p* < 0.05. The prevalence of hyperbilirubinemia was 16.4%( *n* = 30; 95% CI: 11–21.8). Among these jaundiced neonates, 46.7% (*n* = 14) presented with high admission total serum bilirubin levels ranging from 15 to 19 mg/dL, and 26.7% (*n* = 8) required exchange transfusion. Inadequate breastfeeding (AOR 9.51, 95% CI: 1.24–72.67, *p* = 0.030) and neonatal sepsis (AOR 3.71, 95% CI: 1.34–10.28, *p* = 0.012) were the primary independent predictors. The high admission bilirubin levels and significant need for exchange transfusion highlight the urgent need for universal pre-discharge screening and robust lactation support.

## Introduction

Neonatal hyperbilirubinemia results from an imbalance between bilirubin production and clearance, characterized by the accumulation of unconjugated bilirubin pigment in the skin and mucus membranes^1,2^. For most neonates, this is a benign transition; however, excessive levels can cross the blood-brain barrier, leading to irreversible bilirubin-induced neurologic dysfunction or kernicterus^3–5^. Globally, 1.1 million babies develop severe hyperbilirubinemia annually, with 75% of the burden concentrated in sub-Saharan Africa and South Asia^6^. In Ethiopia, where the neonatal mortality rate remains high at 33 per 1,000 live births, jaundice is recognized by the WHO as a critical danger sign^7,8^.

The public health significance of neonatal hyperbilirubinemia in Ethiopia is profound, as it is a leading cause of neonatal morbidity and mortality. Recent systematic reviews indicate a pooled prevalence of 31.6% (95% CI 16.6%–45.3%) in Ethiopia^9^, which is significantly higher than the 24.4% reported across all WHO regions^7^. Furthermore, neonatal jaundice contributes to approximately 10% of neonatal mortality in Ethiopia^9^, with severe cases often leading to bilirubin encephalopathy^6^. A critical public health challenge is late recognition; many neonates arrive at hospitals with admission bilirubin levels of 15–19 mg/dL, increasing the risk of permanent neurological damage^10^. The physiological basis involves higher red blood cell turnover and immature hepatic conjugation enzymes^1,2,11^. Pathological factors—such as sepsis, hemolytic diseases, or inadequate caloric intake—exacerbate this process^12–14^. Furthermore, the condition imposes a high financial burden on families, particularly those earning less than 1,500 Ethiopian Birr (approx. USD 27) per month^15^. While previous national studies focused on urban centers, this study offers unique insights into a specialized setting in the Arsi Zone with a notably high exchange transfusion rate of 26.7%.

## Methods

### Study design

This was an institution-based retrospective cross-sectional study conducted at the ARTH, Ethiopia. The study period spanned from September 1, 2023, to August 31, 2024.

### Study population

The study population included all neonates admitted to the ARTH NICU between September 1, 2023, and August 31, 2024.

### Source and study population

The source population consisted of all neonates admitted to the NICU at ARTH in the Southeast Oromia Region, Ethiopia. The study population specifically included all neonates admitted to the ARTH NICU between September 1, 2023, and August 31, 2024.

### Inclusion and exclusion criteria

- Inclusion Criteria: All neonates admitted to the NICU with a diagnosis of jaundice during the defined study period were eligible for inclusion.
- Exclusion Criteria: To ensure the study focused on neonatal unconjugated hyperbilirubinemia and its primary determinants, the following were excluded:
  - Neonates with cholestasis or conjugated bilirubin levels exceeding 15% of the total bilirubin.
  - Neonates transferred from other primary or general hospitals specifically for exchange transfusion. This exclusion was applied to minimize referral bias and ensure the findings reflect the local clinical determinants and outcomes of the immediate catchment area.

### Selection criteria and sampling

The study population included all neonates admitted to the NICU during the one-year study period. The initial sample size was determined using a single population proportion formula based on a previous 13.3% prevalence^16^.

**Fig. 1:**
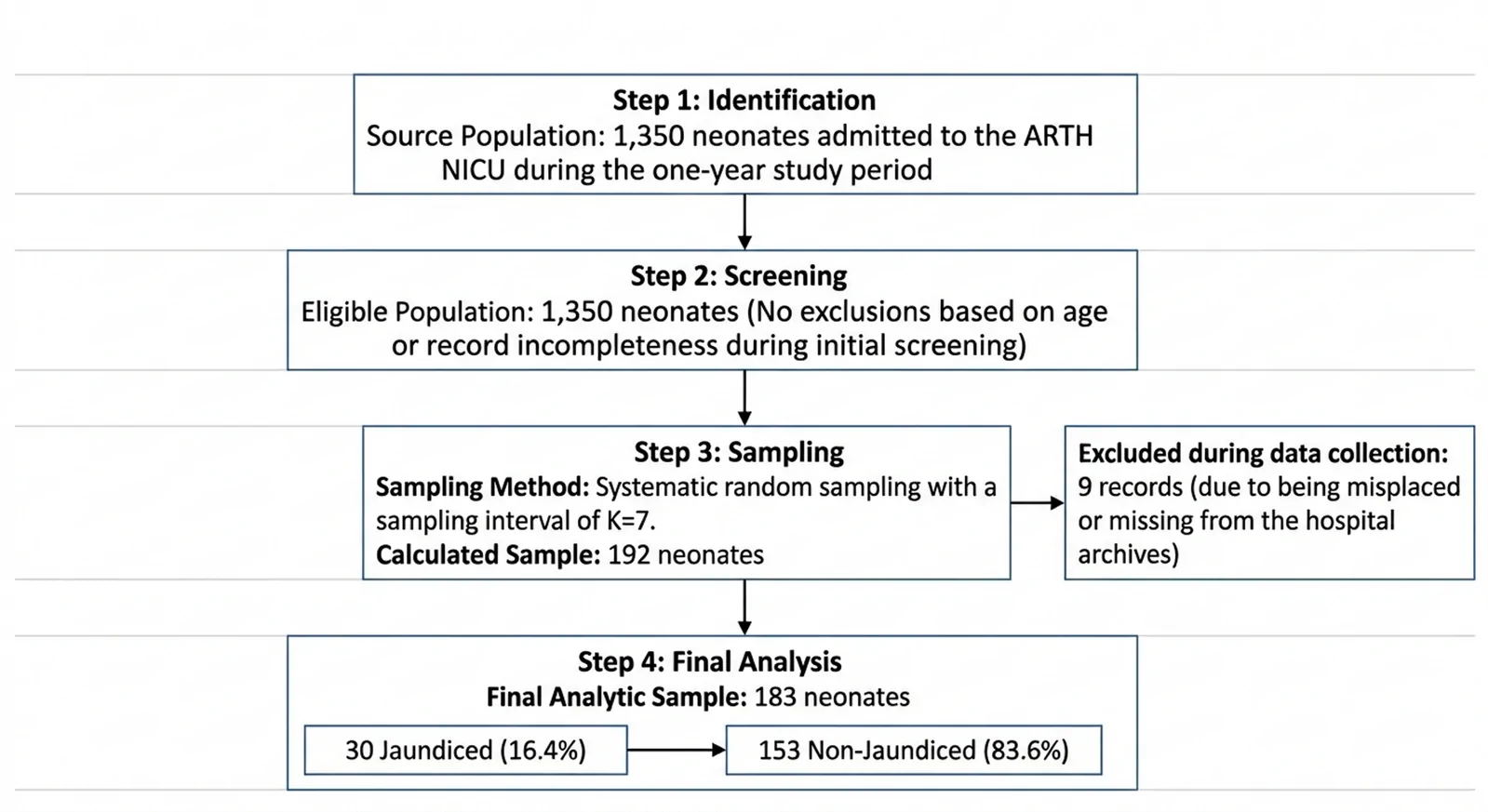
Participant flow diagram showing selection of neonates admitted to the ARTH NICU during the study period, 2024–2025 (*n* = 183).

As shown in the participant flow diagram (Fig. 1), the source population consisted of 1,350 total eligible NICU admissions. We employed a systematic random sampling technique with a sampling interval of K = 7, derived from the ratio of the total admissions (*N* = 1,350) to the target sample size, a method consistent with established retrospective sampling protocols^17,18^. Consequently, every 7th medical record was identified for screening, resulting in an initial selection of 192 neonates. After the exclusion of 9 records due to being misplaced or missing from the hospital archives, a final analytic sample of 183 records was achieved. This final cohort was categorized by jaundice status, consisting of 30 jaundiced (16.4%) and 153 non-jaundiced (83.6%) neonates.

### Variables and definitions

The primary outcome was hyperbilirubinemia (TSB ≥ 5 mg/dL)^3,10^. Independent variables included socio-demographics, neonatal factors (sepsis, prematurity), and maternal factors (blood groups). Inadequate breastfeeding was defined by insufficient intake and low feeding frequency in the first few days of life^1,19,20^.

### Operational definitions

- *Neonatal Hyperbilirubinemia*: Defined as a total serum bilirubin (TSB) level greater than or equal to 85µmol/l (5 mg/dl) as recorded in the neonatal medical charts at ARTH^7,11^.
- *Inadequate Breastfeeding*: (Formerly referred to as breastfeeding jaundice or breastfeeding failure) Defined as jaundice occurring within the first week of life related to insufficient milk intake, characterized by fewer than 8–12 feedings per 24 h, poor latching, or weight loss > 10% of birth weight as documented by the clinical team^1,2^.
- *Breast Milk Jaundice*: Late-onset jaundice beginning after the 4th to 7th day of life, caused by increased reabsorption of unconjugated bilirubin^1,2,20^.
- *Bilirubin Encephalopathy*: A clinical syndrome associated with bilirubin toxicity in the central nervous system, resulting in chronic and permanent neurological sequelae^5,13^.
- *Neonatal Sepsis*: Defined based on the presence of at least two clinical signs (e.g., fever, lethargy, respiratory distress) alongside supportive laboratory findings (e.g., elevated C-reactive protein or abnormal white blood cell count) as per national protocol.

### Data collection procedure

Data were collected using a structured checklist developed by the investigators following an extensive review of relevant literature. To ensure the reliability of the instrument, the checklist was pretested on 5% of the sample size prior to full data collection; necessary adjustments were made by the investigators based on the pre-test findings. The checklist was designed to capture maternal sociodemographic data and neonatal clinical information. To maintain confidentiality, patient names and medical card numbers were excluded from the data collection tool.

### Data quality assurance and bias mitigation

To ensure the integrity of the findings and address the inherent limitations of retrospective data retrieval in resource-limited settings, the research team implemented several rigorous quality control measures. To address potential retrospective bias, the study cross-referenced medical charts with nursing logs and laboratory registers to minimize documentation gaps and ensure the completeness of key clinical variables. The research team further utilized standardized national neonatal management protocols to verify diagnoses such as “sepsis” or “prematurity,” ensuring categorization was based on established clinical criteria rather than subjective judgment. A 5% pre-test of the sample size was conducted prior to full data collection to standardize data extraction and ensure the reliability of the structured checklist. Furthermore, data collection was closely monitored by supervisors to ensure completeness, consistency, and accuracy, with regular review sessions held during the main collection phase to address any discrepancies and maintain a uniform understanding of the clinical records. To mitigate selection and survivor bias, neonates transferred specifically for exchange transfusion from other hospitals were excluded, and the “Age at Admission” was meticulously documented to account for late presentation, acknowledging the study’s limitation regarding neonates who may have died at home or recovered without clinical intervention.

### Statistical analysis

Data were meticulously cleaned to address inconsistencies before entry in to Epi-data version 4.6.0.2 and subsequently exported to STATA version 17 for statistical processing.

Descriptive statistics, including means with standard deviations for continuous variables and frequencies with percentages for categorical variables, were computed to summarize the cohort’s characteristics. To identify determinants of neonatal hyperbilirubinemia, a two-stage regression approach was employed: Bivariable Logistic Regression was used to screen candidate variables with a p-value < 0.25, followed by multivariable Logistic Regression to control for confounding factors. Results were reported as Adjusted Odds Ratios (AOR) with 95% Confidence Intervals (CI). A p-value < 0.05 was used to define statistical significance. The model’s goodness-of-fit was validated using the Hosmer-Lemeshow test, and the absence of multicollinearity among independent variables was confirmed using Variance Inflation Factors (VIF). Finally, the results were presented using a combination of text, tables, and graphical representations.

### Ethical approval and consent to participate

Ethical approval was obtained from the Ethical Review Committee (ERC) of the College of Health Sciences, Arsi University (Protocol/ID: CoHS/R/10/171/2024/2025). Due to the retrospective nature of the study, the requirement for informed consent was waived by the ERC. All patient information retrieved from medical records was handled with strict confidentiality. To preserve anonymity, patient names were replaced with unique identification numbers during data extraction and analysis. All procedures were conducted in accordance with relevant national guidelines, regulations, and the principles of the Declaration of Helsinki.

## Results

### Sociodemographic characteristics

The study included 183 neonates with a slight male preponderance (53%, *n* = 97). A total of 62.8% (*n* = 115)) were admitted within the first two days of postnatal life. Furthermore, 64%,(*n* = 117) of neonates had a birth weight between 2500 g and 4000 g, while 70% (*n* = 128) were born at term (Table 1).

**Table 1:** Sociodemographic and clinical characteristics of NICU admissions, ARTH (*N* = 183).

Characteristic	Category	Frequency (*n*)	Jaundiced Cases (*n* = 30)
Gender	Male	97	14
	Female	86	16
Age at admission	< 2 days	115	12
	2–6 days	44	10
	7–14 days	12	8
	> 14 days	12	0
Gestational age	Preterm (< 37 weeks)	53	7
	Term (37–42 weeks)	128	23
Birth weight	< 2500 g	55	9
	2500–4000 g	117	18
	> 4000 g	11	3

### Clinical magnitude and etiology

**Fig. 2:**
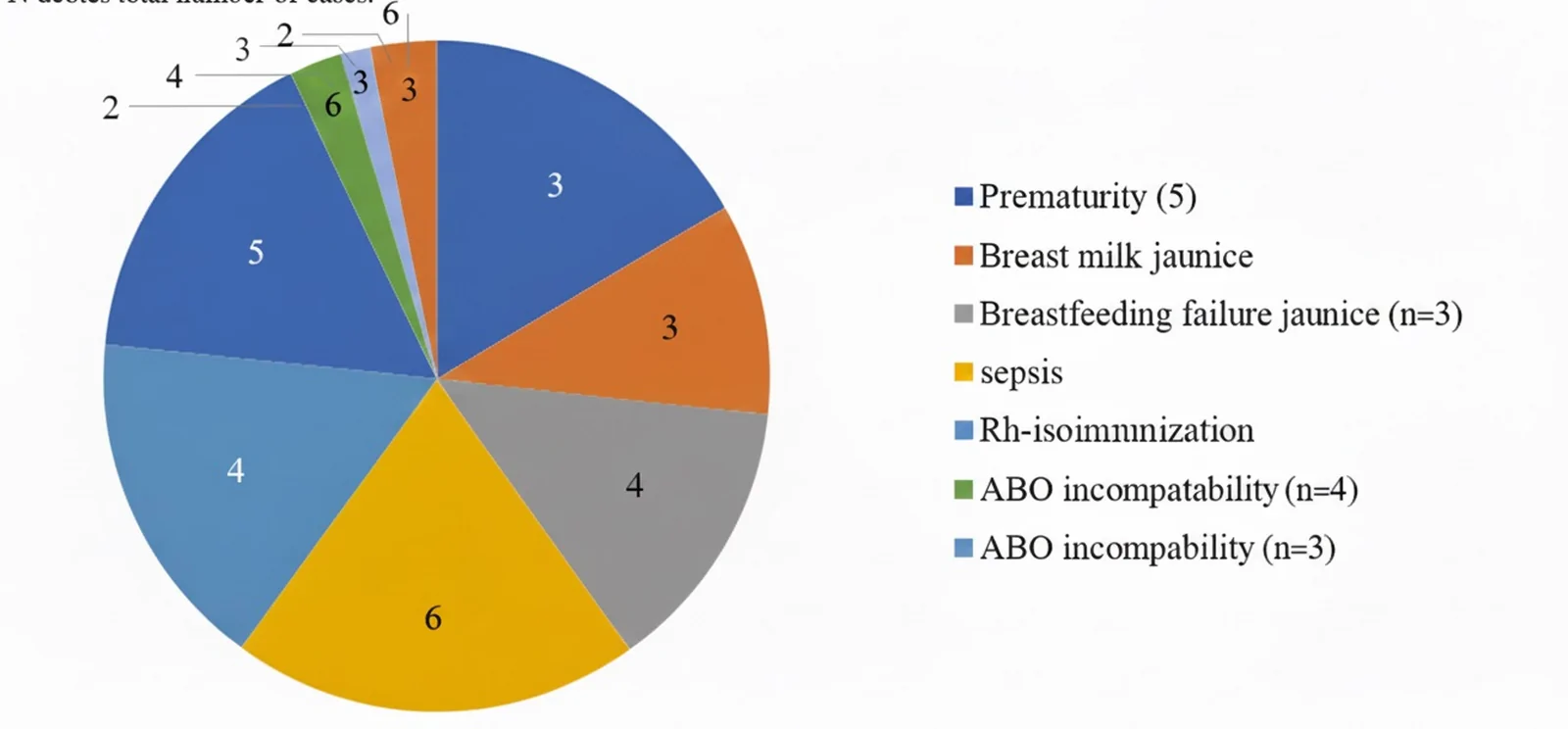
Pie chart illustration of causes of neonatal jaundice among NICU admissions, ARTH. Pie chart distribution of etiologies for the 30 hyperbilirubinemia cases, Sepsis and ABO incompatibility were the most frequent factors.

The prevalence of confirmed hyperbilirubinemia was 16.4% (30/183 cases). As shown in Fig. 2, the primary underlying etiologies identified were sepsis (20%, *n* = 6), prematurity (16.7%, *n* = 5), and Inadequate Breastfeeding (13.3%, *n* = 4). Hemolytic causes were identified in 13.3% (*n* = 4) of the jaundiced cases: three were attributed to Rh-isoimmunization and one to ABO incompatibility.

### Determinants of hyperbilirubinemia

In the bivariable analysis, inadequate breastfeeding, neonatal sepsis, and low birth weight met the criteria (*p* < 0.25) for inclusion in the final model. Multivariate logistic regression revealed that inadequate breastfeeding (AOR 9.51, 95% CI: 1.24–72.67, *p* = 0.030) and neonatal sepsis (AOR 3.71, 95% CI: 1.34–10.28, *p* = 0.012) were the only independent predictors of jaundice (Table 2). Neonates with inadequate breastfeeding were nearly 10 times more likely to develop hyperbilirubinemia (AOR 9.507). Out of 28 jaundiced neonates, 96.4% (*n* = 27) had inadequate breastfeeding, compared to 74.8% (*n* = 116) in the non-jaundiced group. Furthermore, those with sepsis faced a nearly 4-fold risk (AOR 3.707). Specifically, 82.1% (*n* = 23) of jaundiced neonates had a diagnosis of sepsis, compared to 54.8% (*n* = 85) in the non-jaundiced cohort. Other factors, such as maternal age, parity, and mode of delivery, did not show a statistically significant association with the outcome in the final model (*p* > 0.05).

**Table 2:** Bivariate and multivariable logistic regression of factors associated with neonatal jaundice at ARTH, 2021 (*N* = 183).

Variable	Category	COR (95% CI)	AOR (95% CI)	*P*-value
Mode of delivery	SVD	1.97 (0.24–16.08)	0.52 (0.06–4.24)	0.529
	C/S	2.50 (0.26–23.60)	0.36 (0.04–3.40)	0.425
Breastfeeding	Inadequate	9.07 (1.20–68.61)*	*9.51 (1.24–72.67)**	0.030
	Adequate	1	1	–
Birth injury	Yes	1.53 (0.69–3.38)	0.84 (0.18–3.97)	0.829
	No	1	1	–
Neonatal sepsis	Yes	3.79 (1.37–10.49)*	*3.71 (1.34–10.28)**	0.012
	No	1	1	–

ARTH = Asella Referral and Teaching Hospital; COR = Crude Odds Ratio; AOR = Adjusted Odds Ratio; CI = Confidence Interval. Asterisk (*) indicates statistical significance at *p* < 0.05.*.

### Clinical outcomes and management

The clinical severity of neonatal jaundice in this cohort was significant. The mean peak Total Serum Bilirubin (TSB) among jaundiced neonates was 17.2 mg/dL (SD ± 3.4), with values reaching as high as 31 mg/dL. Regarding ABE staging, 13.3% (*n* = 4) of jaundiced neonates presented with moderate to severe ABE, characterized by clinical signs of hypertonia and high-pitched cries.

The data reveals a clear trend of delayed care; 36.7% (*n* = 11) of jaundiced cases presented to the NICU after the critical 72-hour window. Management required intensive intervention, with 73.3% (*n* = 22) receiving phototherapy and 26.7% (*n* = 8) requiring double-volume exchange transfusion. Ultimately, clinical outcomes were positive for 93.4% (*n* = 171) of the total cohort who improved and were discharged, while 3.3% (*n* = 6) deaths and 3.3% (*n* = 6) referrals were recorded.

## Discussion

The 16.4% prevalence of neonatal hyperbilirubinemia at ARTH is higher than the 13.3% reported at St. Paul’s Hospital but significantly lower than the 44.9% at Tikur Anbessa Specialized Hospital^16,19^. This finding is consistent with previous studies conducted in Northern Ethiopia and Southeast Asia^9,12^, which suggest that sub-optimal caloric intake in the first days of life leads to increased enterohepatic circulation of bilirubin. Our findings underscore the urgent need for strengthened early lactation counseling within maternity wards to prevent inadequate breastfeeding.

Additionally, the strong association between neonatal sepsis and jaundice (AOR = 3.71) aligns with recent global evidence, including mechanisms discussed by Hamdi et al. (2025)^19^. Sepsis can trigger hemolysis and impair hepatic bilirubin uptake, complicating the clinical course of neonatal admissions. Given that a significant proportion of jaundiced cases (*n* = 6) presented with concurrent sepsis, integrated management protocols that screen for infection in all jaundiced neonates are warranted.

A key clinical concern identified in this study is the high rate of exchange transfusion (26.7%, *n* = 8), which serves as a proxy for late hospital presentation^11,20^. This interpretation is quantitatively supported by our finding that 36.7% of cases presented after 72 h and the mean peak TSB was 17.2 mg/dL, well above the thresholds for standard phototherapy. When neonates present at such advanced stages, the risk of moderate to severe ABE—observed in 13.3% of our jaundiced cohort—becomes a critical public health issue^10,21,22^.

Unlike some regional studies, maternal parity and mode of delivery were not significant predictors in our setting^21,23^. This variation may be due to differences in the catchment population’s health-seeking behavior or specific clinical protocols at our referral hub. The identified hemolytic cases (*n* = 4), largely driven by Rh-isoimmunization, further highlight gaps in Rh-prophylaxis and the necessity for universal pre-discharge screening to identify at-risk neonates.

## Strengths and limitations

### Strengths

- This study provides localized data for the Arsi Zone, filling a critical gap in regional neonatal health data for Ethiopia.
- The use of multivariable analysis allowed for the isolation of specific independent predictors (inadequate breastfeeding and neonatal sepsis), providing clear targets for clinical intervention.
- The inclusion of recent 2024 and 2025 comparative data ensures the study reflects current clinical trends and publication standards.

### Limitations

- As a retrospective cross-sectional study, it relies on the accuracy of existing medical records, which may involve missing data points common in resource-limited settings.
- The study was conducted at a single referral center; therefore, the findings may not be fully generalizable to primary healthcare settings or home births.
- The sample size limited the ability to perform sub-group analyses on rarer etiologies of jaundice.

## Conclusion and recommendations

### Conclusion

The prevalence of neonatal hyperbilirubinemia at ARTH (16.4%) is a significant public health concern. Inadequate breastfeeding (AOR 9.51) and neonatal sepsis (AOR 3.71) are the primary drivers of jaundice in this population. While overall clinical outcomes were largely positive, the high rate of double-volume exchange transfusions (26.7%) and the presence of moderate to severe ABE (13.3%) indicate that neonates are often identified only after reaching critical bilirubin thresholds.

### Recommendations

- *For Clinicians*: Implement universal pre-discharge bilirubin screening for all neonates to identify at-risk infants before they reach exchange transfusion thresholds.
- *For Hospital Management*: Strengthen lactation support services within the NICU and a postnatal ward, ensuring formal lactation counseling is a mandatory component of care.
- *For Policy Makers*: Integrate neonatal jaundice awareness into standardized Antenatal (ANC) and Postnatal Care (PNC) packages to encourage early hospital presentation.
- *For Researchers*: Future prospective studies should investigate the long-term neurodevelopmental outcomes of neonates who survived ABE or underwent exchange transfusions in this region.

## Data Availability

All data generated or analyzed during this study are included in this published article. Further datasets are available from the corresponding author on reasonable request.
